# Equity and health policy in Africa: Using concept mapping in Moore (Burkina Faso)

**DOI:** 10.1186/1472-6963-8-90

**Published:** 2008-04-22

**Authors:** Valéry Ridde

**Affiliations:** 1 PhD, Department of Preventive and Social Medicine, Medical Faculty, University of Montréal, 3875, rue Saint-Urbain, Montréal, QC, Canada; 2 Centre de recherche du Centre hospitalier de l'Université de Montréal, Montréal, Canada

## Abstract

**Background:**

This methodological article is based on a health policy research project conducted in Burkina Faso (West Africa). Concept mapping (CM) was used as a research method to understand the local views of equity among stakeholders, who were concerned by the health policy under consideration. While this technique has been used in North America and elsewhere, to our knowledge it has not yet been applied in Africa in any vernacular language. Its application raises many issues and certain methodological limitations. Our objective in this article is to present its use in this particular context, and to share a number of methodological observations on the subject.

**Methods:**

Two CMs were done among two different groups of local stakeholders following four steps: generating ideas, structuring the ideas, computing maps using multidimensional scaling and cluster analysis methods, and interpreting maps. Fifteen nurses were invited to take part in the study, all of whom had undergone training on health policies. Of these, nine nurses (60%) ultimately attended the two-day meeting, conducted in French. Of 45 members of village health committees who attended training on health policies, only eight were literate in the local language (Moore). Seven of these (88%) came to the meeting.

**Results:**

The local perception of equity seems close to the egalitarian model. The actors are not ready to compromise social stability and peace for the benefit of the worst-off. The discussion on the methodological limitations of CM raises the limitations of asking a single question in Moore and the challenge of translating a concept as complex as equity. While the translation of equity into Moore undoubtedly oriented the discussions toward social relations, we believe that, in the context of this study, the open-ended question concerning social justice has a threefold relevance. At the same time, those limitations were transformed into strengths. We understand that it was essential to resort to the focus group approach to explore deeply a complex subject such as equity, which became, after the two CMs, one of the important topics of the research.

**Conclusion:**

Using this technique in a new context was not the easiest thing to do. Nevertheless, contrary to what local organizers thought when we explained to them this "crazy" idea of applying the technique in Moore with peasants, we believe we have shown that it was feasible, even with persons not literate in French.

## Background

Amartya Sen [1] tells us that "in any thinking about the fight against poverty, when studying effectiveness or equity, the role of values, from all evidence, is preeminent" (p. 278). Yet, in Africa, analyses of implementation of health sector reforms in recent decades all reach the same conclusion: the equity aspect of public health policies has been neglected and the actors were concerned primarily with the effectiveness of the organization to be set up [2,3]. Our own analyses of public health policies in West Africa, notably in Burkina Faso [4], produce the same assessment. Like Sen, we believe the issue of values, and specifically the way in which equity is understood by the actors, is paramount in understanding the effects of these policies. This is because it is, in fact, quite possible that the failure of the implementation of these policies, in terms of their equity objectives, can be largely explained by the fact that the absence of equity was never seen as a public issue. Yet for any situation to become a public issue many factors must come into play [5], and the question of values is obviously central [6]. Thus it is essential that the concept of equity be understood from an emic perspective. However, understanding this concept is a complex undertaking because equity is a polysemous term whose content depends, at the same time, both on the epistemological position of the researcher and on the social environment of the research. In addition, equity can be studied in terms of many different theories of distributive justice [7-10], that are useful in attaining the ideal of social justice. Here are five examples of different perspectives on distributive justice: (a) the Anglo-American neo-liberalist tradition of equal opportunities [11]; (b) the desire among Swedish politicians to avoid sacrificing equity to efficiency [12]; (c) the rejection in Australia of a policy aimed at maximizing health results if people in poor health must curtail their access to care [13]; (d) the Rawlsian vision of social justice among public health practitioners in Quebec who believe public health should devote much of its resources to specific subgroups of the population (the worst-off; groups of Aboriginal descent; the drug-addicted) who are considered disadvantaged because of socially created injustices [14]; and (e) the rhetorical precedence of egalitarianism among the Mossi of Burkina Faso [15,16], who believe, like their neighbours the Haoussa in Niger, that inequality is constitutive of the social order [17].

To grasp fully the value that the Burkinabè ascribe to equity, it is essential to know the range of definitions they give to the concept. One technique often employed for this purpose is that of concept mapping [18,19]. While this technique has been used in Quebec (Canada) and elsewhere [20], to our knowledge it has not yet been applied in Africa in any vernacular language. Its application raises many issues and certain methodological limitations. In this study, the question of equity was important, but many other empirical elements also contributed to understanding the implementation gap [21]. Also, given the circumstances of this study, only two concept mapping exercises were planned at the outset. Our hypothesis was that the emic perspective of equity was important, but not central and predominant. The key benefit of this technique was that we obtained a group consensus on equity whose outcomes would be analysed and validated by the participants. Contrary to what occurs in most international studies [22], this validation could take place during the research process in the field, rather than waiting for months of analysis in the office and a hypothetical return to the field. Our objective in this article is therefore, on the one hand, to present its use in this particular context, and on the other, to share a number of methodological observations on the subject.

It is a delicate matter to ask participants in a study point-blank what equity means to them, as the term seems so abstract. One dictionary tells us that "equity consists of putting everyone on an equal footing". Thus we would be inclined to think the concept of equity is closely related to that of social justice, since this latter concept consists of trying to achieve equality, understood both as a means and as an objective to be attained. For some, the terms equity and social justice are interchangeable [23]. The notion of social justice will therefore be our conceptual entry point to a better understanding of the social values that surround the implementation of health policies in Burkina Faso.

A study was therefore carried out in one health district using the methodological approach of the case study and of a socio-anthropological field study. The case studied is an international cooperation project of a non-governmental organization (NGO) collaborating in the implementation of a health policy by means of support to State services. The health services are essentially dispensaries managed by nurses. A management committee with members from the village community is partly responsible for the functioning of the dispensary.

The social context is that of the Mossi society of Yatenga. The district's population is very young, the villages are small, and the majority of inhabitants (of Muslim faith and poorly educated) work in farming or herding. The social organization exhibits three fundamental features. The first is a solidarity that, while still important, is deteriorating; 30 years ago, a study of social changes in the Mossi West showed that social cohesion disintegrated over time as principles of authority were diminished, leading to the deterioration of traditional forms of solidarity [24]. Others have noted a tearing of the social fabric, a segmentation of society, increased intolerance and exclusion, as well as an abandoning of socialising [25]. The second feature is a structure that is hierarchical, strict and seeks stability; Pacere [26] went so far as to declare that Mossi organization "denotes total strictness" (p. 93). The third feature is a belief in a "natural" inequality among human beings that is indispensable to social harmony. Badini [27] asserts that "the system of intrinsic inequality among men, as the basis of authority (of the more powerful over the inferior elements) is a major component of Moaga belief" (p.110). Of course, in modern society, customary rights and the foundations of social organization are under discussion and collective identity is undergoing a process of radical transformation [28].

## Methods

To understand the concept of social justice, we have used a number of data collection techniques with the four groups of actors involved in the public policy process. However, this article looks only at the technique of using concept mapping to survey persons belonging to two of the four groups of actors, i.e., members of interest groups and appointed officials [29]. These two groups of actors are considered specialists in health policy (as opposed to individual citizens and elected officials), the former being within the government apparatus and the latter, outside. This is a heuristic division of the groups of actors, given that the same actor might be at the same time, for example, both a nurse and a user of health services. Results having to do with other categories of actors are presented elsewhere [6].

The appointed officials are the nurse managers (*ICP – infirmiers chefs de poste*). They are often obliged to serve as intermediaries between the population, the health services, and the "developers". They are a relatively homogeneous group by virtue of their professional training, but heterogeneous in terms of their region of origin. The heterogeneity of the participants is essential, because it supplies a wide range of actors' viewpoints. Fifteen ICPs were invited to take part in the study, all of whom had undergone training in June 2000 on health policies. Of these, nine nurses (60%) ultimately attended the meeting (mean age 32; in the district for five years; and six years out of the school of public health). While the ICPs have many powers in the dispensaries, the members of the management committees (COGES) made up of village residents are also responsible for implementing activities to improve equity in access to health services. Thus, to gather emic perspectives from members of interest groups, i.e., members of COGES, we used the same tool as for the ICPs. We know, however, that in Burkina Faso the population over age 15 is not very literate in French (13%), yet this technique requires that participants be able to read, because it involves using cards to group together statements describing social justice. In Burkina, over many years, literacy programs in vernacular languages have been offered to villagers. Members of the COGES who were literate in Moore (spoken in the study region) were therefore invited to participate in this second exercise. Of 45 people who attended training in November 2003, only eight were literate in Moore. Seven of these (88%) came to the meeting (mean age 50; farmers and herders; two with no schooling; and five who stopped at the last year of primary school). The participants came from a variety of locations across the territory of the district.

We do not describe here the specifics of the technique [19]. However, since this represents, to our knowledge, its first application in Africa in a vernacular language, we would like to point out certain unique characteristics related to its use in this particular context.

### Generating ideas

In the first phase, we asked participants to explain the term "social justice". To do this, we presented them with the following open-ended question: "In Burkina Faso today, I think the notion of social justice means that...." The session with the nurses was carried out in French. The session with the COGES members was carried out in Moore. Assistance was provided by a teacher (who took part in the first session in French), a sociologist (research assistant) and two members of the Regional Literacy Department. One of these latter two persons wrote on the blackboard the statements produced by the participants to describe social justice, while the other wrote them on forms to be used following the session. It should be stressed that finding a good translation into Moore of the term "social justice" was a delicate matter. Our four assistants engaged in a number of discussions to choose from among what they considered to be the three best options for describing the concept: i) that everyone should be the same (*ti neba faa yi yembre*); ii) whatever unites the country (*sen naagd tenga nen-buiid taaba*); and iii) to not infringe upon others (*n da tab taaba ye*). This last concept was the one ultimately retained by the group. Once written on the board, each statement was restated orally and validated by the participants to avoid any interpretation on the part of those charged with writing the Moore on the board.

### Structuring the ideas

In the second phase, still at the individual level, participants dealt with the statements in two ways. First, they grouped them into as many piles as needed "*in a way that makes sense to them*" [[18], p.7]. Concrete examples were provided to help them in this task. The individual piles were then numbered and given a name by each person. In a subsequent step, each person attributed to each statements a score for importance, on a scale of 1 to 5 (Likert style). To help the COGES members in this second task, we gave the example of Guinea Worm Disease, letting them attribute a score of 1 to 5 to five causes of this disease that were known to the villagers.

### Computing maps

The third step was carried out only by the researcher, who had, up to that point, not handled the data in any way. In this step, the statements, with their identification numbers, scores and attributions to a numbered group, were collected using a specific software program. The statements were translated into Moore by two persons from the department of literacy service, along with the sociologist, with the aim of reaching a group consensus on the terms that would work in French. This step allowed us to produce a first concept map, in the geographical sense of the term. Two types of statistical analysis were carried out. The first, multidimensional scaling, consisted of a multivariate analysis that had the advantage of allowing for each statement to be positioned in relation to the others in accordance with the strength of association. This strength of association is determined by the number of times the statements are found within the same pile by many participants. Thus, the most strongly associated statements were very near each other on the graph. The second analysis, hierarchical clustering, created clusters of elements having similar concepts. The researcher decided on the number of clusters based on a heuristic perspective, i.e., following "a subjective inspection of the different levels of analysis" [[30], p. 17]. The objective of this cluster analysis was to produce a map of clusters that would provide a statistical perspective of the group of participants, based on the groupings created by each individual. In this way, the statistical analysis supported consensus-building on the semantic significance of the clusters in the following step. Also, because the participants had scored each statement, it was possible to assign relative values to each of the conceptual categories and to each statement. The average of the scores of all the statements in a cluster is the mean value of the cluster (see Figure legends 1 and 2). The mean value of each statement for all participants is presented in Additional Files 1 and 2.

**Figure 1:**
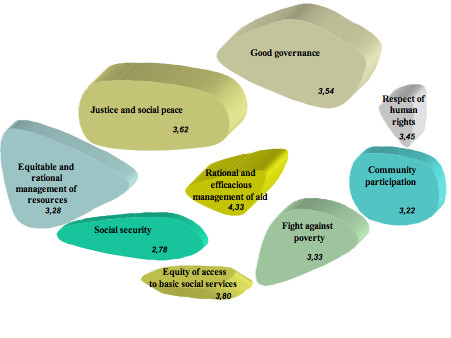
**Concept Mapping and Social Justice for ICPs (n = 9)**. Note: Initial quotes in French. Numbers represent the mean of importance for each cluster (from 1 to 5). The most important clusters have a 3D depth of 100 points, the next 90 points, etc...

**Figure 2:**
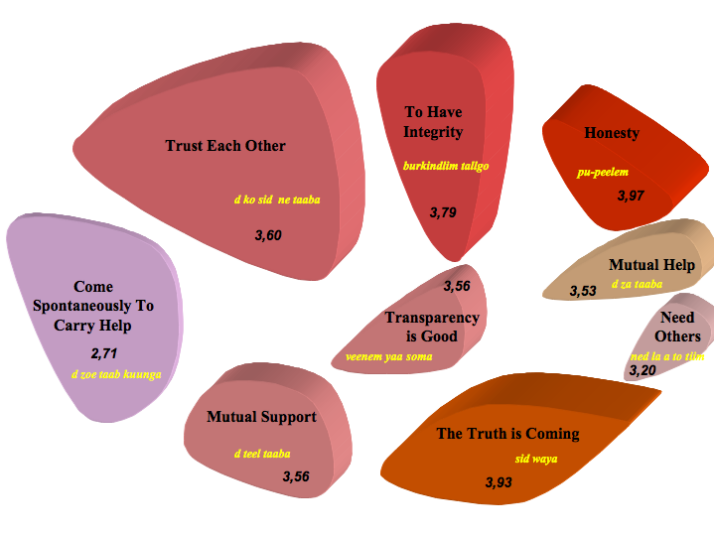
**Concept Mapping and Social Justice for COGES members (n = 6)**. Note: Initial quotes in Moore. Numbers represent the mean of importance for each cluster (from 1 to 5). The most important clusters have a 3D depth of 100 points, the next 90 points, etc...

### Interpreting the map

The fourth step goes back to the participants (see Additional files 3 and 4). Based on concepts that had been regrouped into clusters by means of the statistical analyses, participants had to name – this time with group consensus – the different conceptual sets in accordance with the statements they encompassed. It was necessary to reach consensus because the participants did not see in this visual representation the same clusters that they had produced individually. After a few minutes of individual reflection, the various propositions were noted on the board to help participants reach consensus. Participants were also free to decide that the statistical analysis was not relevant and that a statement did not belong in a certain cluster and should in fact be moved to another. Statements were moved only when requested by more than one person. When there were different ideas about where the statement should go, a vote was held to determine the majority opinion. They could also decide to regroup certain clusters into more congruent "regions".

Our presentation of this technique of Concept Mapping demonstrates that we elected to use it because its relevance has less to do with statistical analysis than with the process of producing and validating the results. This is an advantage described by others who have used the technique [31,32]. It is the entire process, not only the statistical analyses, that is relevant, and particularly the graphical representation it makes possible. This rigorous process produces results that make sense for the participants because their validation of the analyses is predominant. Producing data that the participants consider to be valid, without sacrificing the rigour of the statistical analysis, is thus the central criterion of the relevance of this methodological choice.

This study was approved by the Research Ethics Committee of Université Laval (2003-159) in Canada and by the Health Research Ethics Committee in Burkina Faso (2003-017). After the participants were informed of the advantages and disadvantages of participating in the study, they signed a form approved by these committees, giving their free and informed consent.

## Results

The results presented here are organized according to the two categories of actors who participated in the data collection process. Because the focus of this article is on methodology, only the main results needed for a discussion of the concept mapping method are presented. A more detailed presentation of the results can be found elsewhere [4,16,21].

### The appointed officials

The ICPs expressed 80 statements. In studying these, one of the most important observations, in our opinion, was that these officials brought up fundamental processes associated with social justice for which the responsibility for implementation was most often located far outside their own sphere of activity. For most of them, social justice is perceived as attributable not so much to individual behaviours, but rather to the way the whole society, and particularly the State, operates. Thus, the nurses put forward relatively abstract ideas such as *human rights*, *democracy*, or even *good governance*. With the exception of a few specific items, these three latter concepts seemed to us to outweigh, for the nurses, all other propositions relative to social justice in Burkina Faso.

The consensual labelling of the clusters (devised as conceptual groupings) by the ICPs provided the results presented in Figure 1 (see Additional file 1).

If we look at the importance attributed to each of these nine concepts for explaining the idea of social justice, we note that three clusters of concepts stand out. Two clusters are made up of a single concept, the most and the least important, and the seven others are ranged between these two.

While the process of managing public aid for development did not actually appear in the individual groupings, the nurses attributed to it the greatest importance in describing social justice. It was the synthesis of individual groupings using statistical analyses that ultimately generated this new and distinctive category. A useful feature of concept mapping, in its participative aspect, is that it gives participants a chance to validate such an analysis, which they did gladly. Thus, to the statements regrouped by the statistical analysis, they assigned the label: *rational and efficacious management of aid*. This way of seeing things, on the part of the ICPs, is probably best explained by the fact that they experience and perceive every day how this dimension of social justice is distorted. They are partly responsible for this situation, but this does not prevent them from placing the concept at the top of the pyramid. The fact that the most important concept (4.33) is at the centre is probably not a statistical accident. We might offer the hypothesis that the existence of all the other concepts depends upon this one. In other words, without rational and efficacious management of international aid, there is no good governance, no justice, no social security, and no fight against poverty. We would add that this concept is also very close to the way the State works, since it participates in the management of aid (although not alone, which is what justifies the existence of this unique category).

The concept which least represents the ICPs' idea of the concept of social justice is that of *social security*, the only one below the mean of three points. Conceptually – and this comes through in the map – this category is similar to the one that touched upon equity of access to health services. However, after careful discussion, the participants decided not to merge these two concepts and to identify social security as a dimension in its own right. This decision is most probably attributable to a desire to dissociate health services from social services in general and to adopt a broad vision of the nature of social security. In fact, on closer examination of the statements that make up this concept, we observe that the ICPs included, within social security, the concepts of security of employment and of income. The notion of social security must therefore be understood in the broadest sense of social protection. It is hardly surprising that they gave this category the lowest score (2.78), because even if the nurses believe it is fundamental to social justice, they are nevertheless realists and know it is probably the most difficult objective to achieve at this time, in the current context.

Between these two extremes, which stand out by a wide margin, seven concepts were provided by the nurses to describe social justice. The second position, in order of significance, is allocated to *equity of access to basic social services*. We must point out here that we unfortunately did not discuss with the participants their definition of "equity". If they use this term, it is most likely because it is in some way a common expression. It seems to us this concept means, above all, that access to services should be the same for everyone and that there should not be any positive discrimination for one group or another. In this category, equity for the indigent received the lowest score (2.89), in contrast to access for all (4.67).

After this comes the cluster of *justice and social peace*. The map shows this concept to be conceptually very close to that which follows immediately, in decreasing order of significance, which is *good governance*. On closer examination, it is clear they could have been merged without any real change to the importance attributed to this dimension of social justice (the mean would have gone from 3.62 to 3.57). While these two concepts are not the most significant in terms of weight, they are in terms of the number of statements. Together they represent almost 40% of the total statements produced, with good governance having the greatest number. The *equitable and rational management of resources *and the *respect of human rights *do not "weigh" as much but are placed in the same region of the concept map, as two extensions of justice, social peace and good governance. Resource management seems to be understood by the participants from a distributive perspective, since they mentioned primarily issues related to equitable distribution (of salaries, equipment, honours, allocations) – that is, in terms of wealth, of risk, of abilities, and of needs. At the heart of the map, these five concepts ultimately form an arc of a circle that is relatively dominant and consistent, all being very much linked, in our opinion, to the role of the State.

Finally, two concepts received very similar mean scores: *fight against poverty *and *community participation*. These two concepts are very connected. The community is associated with poverty. Given that poverty in Burkina remains a phenomenon that affects the countryside more than the cities, it could be inferred that the ICPs link the concept of community with that of rurality and poverty. When they think of community, they think mainly of the poor and the peasants.

All in all, what is most interesting in looking at the positions of the nine clusters is that three entities seem to emerge. The State, whose essential role is to guarantee the benefits represented by the four concepts located at the top of Figure 1, seems to control a population concerned with the four others located at the bottom. Between the two, as a concept resulting from the process of their conjunction, we find the management of aid. Beyond the clichés of "North-South" or of the "developers" and the "developed", it may be that the participants are telling us that the manner in which aid is managed is depends very much on this conjunction and is embedded in the particular relationships that connect populations with government.

### Interest groups

The participants produced a total of 59 different statements. They particularly stressed *honesty*, *truth*, and *transparency*, mainly terms opposed to corruption or to the misappropriation of aid. Political action, if it corresponds to these prerequisite qualities, represents one modality of social justice. The State or the government has an undeniable role to play in social justice, according to the peasants. No clear idea emerges from the analysis as to the society's ability, as a whole, to act collectively and to guarantee equity. On the one hand, solidarity is put forward, while on the other, there appears to be little confidence in the efficacy of collective action. Notably (among other things), the members of the management committees made no connection between social justice and improvement of living conditions for subgroups of the population commonly described as vulnerable (women and indigents), except perhaps for children.

The consensual labelling of clusters for the interest groups produced the results presented in Figure 2 (see Additional file 2).

This map, characterized by nine different concepts expressing the idea of social justice, appears to be split into two entities: one having to do with values, and the other, with action.

If we simply analyze those concepts with the highest scores after the statistical analysis and the labelling by participants, *honesty *and *truth *emerge clearly, and *integrity*, coming in third place, remains very nearby in terms of importance. However, adding to these three concepts the next two in decreasing order of importance, i.e. *self-confidence *and *the transparency is good*, appears to reveal a conceptual entity very clearly oriented in the same direction, i.e., that of values. These five concepts are quite near each other on the map and we observe a T-form where the three categories at the top of the map express the same idea. For the COGES members, these five concepts would be the fundamental values of social justice. Although the peasants, in their definitive statements, did not try to connect these designations to individuals or groups, or even organizations, we can see, on looking more closely at the wording of the clusters, that these values relate to all the concepts.

The second entity, within which the other four concepts can be grouped together, highlights action as a key component of social justice. For the peasant members of the management committees, social justice is a dual notion, with values on the one hand and action on the other. It is through intervening that we are able, they tell us, to achieve a certain level of social justice. What is interesting in the labels attributed by the participants is that these actions seem to arise foremost out of collective considerations. Thus, people must *support each other *and *help each other*. Then, along the same lines, the two concepts designating action that are ranked lowest in order of importance are those that can be associated with individual behaviours. We can thus establish that *needing others *and *spontaneously helping *individuals seem to carry less importance for COGES in the conceptual definition of social justice. While these orders of importance have to be considered, we must not forget that this map should also be read from a global perspective, taking into account the spread of the concepts, to understand equity from the emic perspective. In addition, we should add that the concept with the least importance is also the one with the highest bridging index. It is also the concept that garnered the least consensus among the participants when it came to allocating statements to it. The statement with a perfect disagreement score (1.00) with regard to its being positioned in one category or another is that aimed at *aid for those most in need*. It seemed to be the odd one out, with the participants not knowing whether it belonged with values or action or, quite simply, with social justice.

## Discussion

One advantage of having used the same statistical and visual technique for collecting the perspectives of both the appointed officials and the interest groups is that it allows comparison. This is an endeavour that has not yet been sufficiently explored [33]. A comparative discussion of the results, as well as of the convergent and divergent elements of the two groups of actors and of certain specific aspects, is presented elsewhere [4]. The purpose of this article being to contribute to the development of knowledge related to the use of this technique in a new context, we want to focus in this discussion on the methodological limitations of our study and of the technique used.

### Foreign observer status and validity of the data

To limit certain biases, particularly those related to our status as foreign observers, we prolonged our stay, which was the second one, in the field for seven months. This relatively long period made it possible, in part, to reduce "disturbances occasioned by [our] presence" [[34], p.77] and to increase our chances that "the study [can] elicit the realities that it aims to record" » [[35], p.1410]. Moreover, the results of this study have been shared and discussed on many occasions. First, this was done at four scientific conferences, of which one was in Burkina Faso and another in Canada in the presence of a representative of the *Association burkinabé de santé publique *(Burkina Association for Public Health). Then, 14 months after our departure from the field, two workshops were organized in Burkina Faso with 30 persons involved in the issue being studied. Their comments and questions were useful in challenging and deepening our interpretations.

As to the transferability of our conclusions with regard to social justice, it must be stressed that the development of data from concept maps should, in no case, be used to generalize results to all the inhabitants of the study region, and even less those of the country as a whole. It must be remembered, for instance, that for reasons of context, we only questioned men (in contrast to other data collection techniques used in this study). It was obviously beyond our scope to seek information saturation, whether theoretical or empirical [36]. The primary objective of this data collection and subsequent analyses was to provide us with some indication of emic perspectives of the concept of social justice among these given groups of persons, and no more than that. The advantage of this technique is that it revealed a general orientation of distributive justice that appears similar to the egalitarian model [16]. The people we questioned were not ready to compromise the stability and social peace of the whole (according to the famous principle of holism [37]) in order to act on behalf of the underprivileged). The Mossi, in effect, tend toward social pacification and a strict hierarchical organisation, as confirmed in earlier studies [27,38].

Our study is not focused solely on social justice. It is meant to be a study of the implementation of health policies, at the heart of which social values occupy a predominant but not exclusive position [21,39]. The complexity of the object of the study requires, for its analysis, a broader approach than just examining social values. Understanding values is only one of nine factors that are important for making sense of the fact that the situation of excluding indigents from access to care was not perceived as a public issue [4]. Our simple analysis of the concept of social justice is part of this much larger theoretical imperative and comes under the resources allotted to the present study.

### The limitations of asking a single question in Moore

We must say a few words here about the methodological limitations raised by the use of a data collection technique that is based on statements produced in response to a single question posed to the participants. This concern arises more commonly among experts in quantitative methods using standardized questionnaires – "If one question works, why ask several?" asks Anne Bowling [40] – than among those using a socio-anthropological approach that presupposes the analysis of mainly qualitative data. Contrary to most qualitative techniques used to obtain the perspective of actors, this mapping technique (a mixed-method) offers no margin of manoeuvre in terms of questioning. This is not common for users of such an approach because, most often, researchers have a certain amount of freedom and a lot of flexibility in their interactions with the participants. Therefore selecting the single, definitive question is strategic. This limitation is mentioned by others who have used the technique [41]. This is especially true in our situation, where the sentence was translated, because "the triad (researcher-interpreter-informer) does not transform the ethnological situation into a farce, but rather into a potential source of compromise, secrecy, or obviously, misunderstanding" [[42], p.67]. Regarding translation, there was no comprehension problem among the nurses, because the question was asked in French. But for the COGES members, it was another story entirely. As it happened, the translation of the question on social justice posed to the peasants by the four local experts referred to how humans interact among themselves: "*to not infringe on others*". While this translation undoubtedly oriented the discussions toward social relations, we believe that, in the context of this study, this sentence has a threefold relevance.

First, this translation was understood, in the context in which it was conceived and at the time it was produced, as being the nearest expression of the concept of social justice, according to these local experts, who endeavoured to transpose an idea expressed in French into a sentence that would be comprehensible in Moore. We have no particular reason to believe the sentence proposed by these four persons is any more or less exact than other attempts at translation. Nevertheless, using this translation certainly represented a gamble and embarked us on a venture that was fraught with potential pitfalls. As in the experience of Fassin [43], who analyzed constructions of the intolerable in so-called traditional societies where the word "intolerable" does not exist, we doubtlessly took "a specific epistemological risk", since "abstraction of the concept used and its importation from a different world involves a significant element of interpretation" (p. 22) Sources of interpretation error on the part of the researcher are in any case relatively fewer in the mapping technique, given that participants play a certain role in data analysis, particularly when they name the clusters formed after statistical processing. This is one of the advantages of this technique, which makes it possible for the actors themselves to use whatever vocabulary they prefer [44]. Of course, the words used are not neutral. They are embedded in social structures and denote thought systems and processes of "internalization". With regard to translation, others before us have tried to translate the notion of social justice into Moore, and for lack of any satisfactory expression, decided to defer the translation and to get around the problem by resorting to operational questioning [45], such as we did in focus group with individuals. We believe that our having taken this risk will ultimately prove useful to other researchers in providing a better translation, if needed.

Second, the peasants were advised by the four translators, at the start of the mapping exercise, of this translation problem. The participants knew the question that had been asked in French to the group of nurses, and they understood it because, while not literate in that language, they knew it well enough to appreciate the challenge. However, to facilitate their discussions, the question had to be posed to them in Moore.

Third, we believe it is possible, from statements made about the nature of social relations, to make inferences on what social justice represents for the participants. For Sen (2000), "norms and notions related to justice determine behaviours" (p.272), and so human behaviours are thus related to justice. Doesn't the manner in which we interact with each other, or how some people infringe upon others, or how, for example, we think about subgroups of population, provide evidence of our concept of social justice? The fact that no one seems to want to favour certain subgroups to the detriment of others (according to the principles of holism and hierarchy highlighted by Louis Dumont [37]) provides some indication of the conception of social justice. Social relations are evidently not the only characteristic of social justice. However, given the context of this study and our need, for the mapping exercise, to come up with a translation in the language spoken by the participants, the choice we made was the most appropriate in terms of responsiveness. Nevertheless, we must admit that, as presented in Moore, the question could lead the participants into a deliberation oriented more toward procedural justice (distribution of means) than distributive justice (distribution of the ends). This might explain, for example, the absence of any consideration of health status in the statements made by participants to describe social justice.

### Limitations transformed into strengths

Faced with these few limitations, we believed it was essential to resort to the focus group approach. Use of this data collection tool was not originally planned in the research protocol. We used it mainly for two reasons.

The first reason has to do with the technique and its limitations when it comes to exploring deeply a complex subject such as social justice. Although at first we thought concept mapping exercises with individual officials and interested persons would be sufficient for comprehending the notion of social justice, we later concluded this was not the case. Consequently, after the first mapping exercise with the nurses, we felt we needed to probe more deeply into the understanding of concepts of social justice. To do so, we allowed the social actors to talk at length about their conception of equity. While concept mapping offers certain advantages in terms of data analysis related to individual interviews (particularly with regard to coding issues [33], researchers doing mapping sometimes turn to data triangulation using complementary qualitative methods [46].

The second reason, which is a beneficial aspect of this methodological limitation, has to do with the object itself of research on equity and health policies. The importance of the issue of values (in this case, social justice) underlying the implementation of health policies was not actually *revealed*, because we had planned to study it, but rather *accentuated *following the mapping exercises. The application of this technique led us to really consider the crucial importance that must be attributed to values and to local concepts of social justice when analyzing public policies. The preliminary results showed that values provided a direction that was very relevant *a priori *in explaining the exclusion of indigents in the organization of the health care system. Also, we could not content ourselves only with the perspectives of the officials and the interest groups, but also had to question individuals (the perspective of elected officials being studied through document analysis). Nevertheless, for reasons having to do with participants being required to write, but also because of the methodological limitation mentioned above, we decided not to use concept mapping for the category of individuals. For this category, we adopted the focus group approach [47]. We asked certain questions which allowed us to comprehend the nature of the theory of justice in action. For example, participants were presented with a case study where they had to distribute a donation of millet in the village. In addition, to counteract the problem mentioned earlier regarding the translation of the notion of social justice, this notion was operationalized in group interviews by means of open questions focused on social and health inequalities. The absence of social justice in a given society implies the creation of social and health inequalities. In other words, we wanted to know and understand individual reactions to the existence of such inequalities by making reference to the daily lives of villagers. This was a useful strategy for uncovering the actors' perspectives on the notion of social justice. Consequently, four discussion groups were convened. The results complemented those coming from the mappings and largely confirmed, through this kind of data triangulation, the analyses of social justice, and particularly of the intense desire for social peace [16,21].

## Conclusion

Using this technique in a new context was not the easiest thing to do. We have mentioned some of the limitations and strengths in this respect. Nevertheless, contrary to what local organizers thought when we explained to them this "crazy" idea of applying the technique in Moore with peasants, we believe we have shown that it was feasible, even with persons not literate in French. It is clear that in such a context, the quality of the translation is the cornerstone of the analysis, as well as of its credibility. However, no individual persons nor data collection techniques can have the last word, nor even the truth. Beyond the limitations inherent to concept mapping, its application under these conditions has again shown the importance of different forms of triangulation (of data, of informers, of techniques, of translators, of analysts) for understanding a concept as complex as that of social justice. The process of validating the results of this analysis was carried out in several stages. At the very the least, we strove to achieve a certain degree of consensus on the emic perspectives of social justice in the concept mapping, first with the translators, and then – and most importantly – with those producing the statements. Producing data, analyzing them, and presenting their interpretation to everyone involved was all done within the space of one week. This is a great advantage, especially in the context of international studies where the researcher returns quickly to his or her country and comes back, if at all, many months or years later [22]. Subsequently, discussions were organized at the time of data collection on the concept of social justice with other informers and using other techniques. Finally, this validation of our interpretations was strengthened by a comparison with the earlier scientific literature, by consideration of critical reviews of our writings from other scientists, and ultimately, upon the release of our research results two years later, by the point of view of stakeholders in Burkina Faso.

## Competing interests

The author declares that they have no competing interests.

## Authors' contributions

VR planned the design, carried out the research, collected and interpreted the data, and wrote the manuscript.

## Pre-publication history

The pre-publication history for this paper can be accessed here:



## Supplementary Material

Additional file 1 **Appointed officials' statements and clusters**. The data provided represent the list of appointed officials' statements and clusters (including means scores and bridging index). Click here for file

Additional file 2 **Interest groups' statements and clusters**. The data provided represent the list of interest groups' statements and clusters (including means scores and bridging index). Click here for file

Additional file 3 **Validation and labelling of the clusters in Moore**. Picture of participants and moderators validating and labelling the clusters in Moore. Click here for file

Additional file 4 **Participant cluster labelling in Moore**. Picture of one participant labelling clusters in Moore. Click here for file
