# Supplementary material for: Equity and health policy in Africa: Using concept mapping in Moore (Burkina Faso)
Source: BMC Health Serv Res. 2008 Apr 22;8:90. doi: 10.1186/1472-6963-8-90 (PMC2386119; doi:10.1186/1472-6963-8-90)
Supplement: Additional file 1 — Appointed officials' statements and clusters. The data provided represent the list of appointed officials' statements and clusters (including means scores and bridging index). [file 1472-6963-8-90-S1.pdf]

#	STATEMENTS	MEANS OF STATEMENTS	BRIDGING INDEX	Name of cluster
**Cluster 1**	**Mean of cluster**	**4,33**	**0,31**	**Rational and efficacious management of aid**
65	The donations of partners should not be diverted	4,56	0,34	
64	The donations of partners should be rationally managed	4,44	0,31	
78	Aid should actually reach its intended beneficiaries	4,00	0,29	
**Cluster 2**	**Mean of cluster**	**3,80**	**0,37**	**Equity of access to basic social services**
39	Each person should have access to basic social services	4,67	0,34	
49	Everyone should have access to education	4,22	0,33	
56	All the elements of the Primary Health Care should be applied	4,11	0,40	
2	The sick should receive equitable treatment	3,11	0,41	
3	The poor should be looked after equitably	2,89	0,36	
**Cluster 3**	**Mean of cluster**	**3,62**	**0,54**	**Justice and social peace**
27	All social classes should be involved in decision-making	4,67	0,77	
37	Everyone should be judged according to the same rights	4,67	0,68	
48	Corruption should be abolished	4,22	0,37	
32	All citizens should be subjected to the law	4,00	0,59	
38	Ethnic discrimination must be avoided	3,89	0,49	
25	Work should be paid according to the effort expended	3,89	0,35	
41	Xenophobia should be abolished	3,56	0,51	
12	We should respect each other's integrity	3,22	0,40	
43	The State should be secular	3,22	0,75	
42	Religious discrimination should be avoided	3,00	0,37	
17	Services should be decentralized	2,89	0,70	
61	Authors' rights should be recognized	2,22	0,52	
**Cluster 4**	**Mean of cluster**	**3,54**	**0,12**	**Good governance**
1	There must be good governance	4,56	0,00	
50	We should fight against impunity	4,56	0,31	
36	Avoid wheeling and dealing in public affairs	4,22	0,25	
72	Avoid abuses of power	4,11	0,08	
73	Decisions must not be taken arbitrarily	4,11	0,22	
67	Political power should alternate between parties	3,89	0,00	
35	Avoid giving preferential treatment to influential parties	3,67	0,00	
70	Elections should be free and transparent	3,67	0,13	
13	Public matters must be managed rationally	3,67	0,30	
31	There should be strict monitoring of government activities	3,67	0,38	
23	There should be a strong democracy	3,67	0,05	
24	Everyone should have the same right to vote	3,44	0,08	
33	The democratic process should be fluid	3,11	0,05	
71	Political mandates should be short	3,00	0,09	
51	There should be a political opposition party	2,89	0,00	
22	There should be a multiparty system	2,78	0,00	
30	Avoid creating political parties along ethnic lines	2,78	0,13	
80	The Faso Mediator should carry out his role	2,78	0,11	
34	All parties should be allowed access to the media	2,67	0,13	
**Cluster 5**	**Mean of cluster**	**3,45**	**0,38**	**Respect of human rights**
4	There must be freedom of expression	4,33	0,31	
8	Human rights must be promoted	4,22	0,41	
57	The right to strike should be respected	3,78	0,43	
5	People and goods should circulate freely	3,56	0,37	
9	Rights should be assured by law	3,56	0,41	
45	People across the country should feel free	2,78	0,37	
11	There should be equality of the sexes	2,67	0,34	
75	Children should be accepted regardless of their sex	2,67	0,36	
**Cluster 6**	**Mean of cluster**	**3,33**	**0,34**	**Fight against poverty**
74	Everyone should have a dwelling	3,78	0,28	
52	Everyone must have a minimum to live on	3,56	0,44	
47	Everyone should be free to work the land	3,00	0,28	
46	Landowners should be treated equally	3,11	0,30	
26	Remove barriers between rich and poor	3,11	0,37	
21	The poor should have an acceptable living	3,56	0,34	
20	We must fight against poverty	3,44	0,34	
16	There should be solidarity in the distribution of the country's resources	3,44	0,31	
10	Social classes should be abolished	3,00	0,37	
**Cluster 7**	**Mean of cluster**	**3,28**	**0,65**	**Equitable and rational management of resources**
6	There should be proper distribution of the fruits of everyone's labours	4,78	0,41	
66	Pay levels should reflect the risks of the job	3,89	0,60	
60	Honorary titles should be awarded based on merit	3,78	0,70	
15	There should be equitable geographical distribution of resources	3,44	0,79	
40	The Minister of Social Action should actually carry out his responsibilities	3,22	0,80	
77	Equipment should be distributed according to needs	3,11	0,68	
68	Officials should be appointed in accordance with their competencies	2,89	0,53	
53	Pensions should be ensured for all services rendered to the State	2,67	0,55	
69	Officials should be appointed in accordance with the needs	2,56	0,48	
58	Non-strikers should not have the right to measures granted to strikers	2,44	1,00	
**Cluster 8**	**Mean of cluster**	**3,22**	**0,39**	**Community participation**
7	Take into account the aspirations of the people before taking decisions	4,22	0,32	
29	Consider the opinions of the poor	3,78	0,38	
28	Allow the poor to express themselves freely	3,78	0,45	
14	There should be collective participation in decision-making	3,78	0,43	
19	The community should get involved in the life of service	2,44	0,43	
18	Decisions should be taken at the local level	2,44	0,37	
62	People should be free to create their own employment	2,11	0,33	
**Cluster 9**	**Mean of cluster**	**2,78**	**0,52**	**Social security**
76	Workers should have the means necessary to carry out their jobs	3,44	0,59	
55	Social security should be strengthened	3,33	0,41	
63	We should improve access to credit for the poor	3,22	0,33	
79	Workers in the informal sector should have status and rights	3,11	0,38	
59	Peasants must be able to sell their products	2,78	0,89	
44	Women should be emancipated	1,89	0,44	
54	Unemployed graduates should be remunerated	1,67	0,63	
