# Supplementary material for: Equity and health policy in Africa: Using concept mapping in Moore (Burkina Faso)
Source: BMC Health Serv Res. 2008 Apr 22;8:90. doi: 10.1186/1472-6963-8-90 (PMC2386119; doi:10.1186/1472-6963-8-90)
Supplement: Additional file 2 — Interest groups' statements and clusters. The data provided represent the list of interest groups' statements and clusters (including means scores and bridging index). [file 1472-6963-8-90-S2.pdf]

| # | STATEMENTS | MEANS OF STATEMENTS | BRIDGING INDEX | Name of cluster |
|---|---|---|---|---|
| **CLUSTER 1** | **Mean of cluster** | **3,97** | **0,24** | |
| 4 | Tell each other the truth | 4,33 | 0,16 | |
| 18 | Give everyone a chance to speak | 3,67 | 0,26 | Honesty (pu-peelem) |
| 32 | Ensure that aid reaches its destination intact | 3,50 | 0,32 | |
| 22 | Justice must be transparent and independent | 4,17 | 0,26 | |
| 40 | The forces of order must operate as they should | 4,83 | 0,14 | |
| 42 | Avoid the concurrent holding of multiple positions of responsibility | 3,33 | 0,32 | |
| **CLUSTER 2** | **Mean of cluster** | **3,93** | **0,10** | |
| 5 | Work and eat together | 2,50 | 0,14 | |
| 34 | Ensure there are sufficient medications for the whole population | 3,33 | 0,13 | |
| 47 | Directors should share responsibilities with their subjects | 4,17 | 0,10 | |
| 38 | Ensure that aid gets to its destination | 4,50 | 0,09 | The truth is coming (sid waya) |
| 49 | Local officials must work for the benefit of the whole village | 4,33 | 0,09 | |
| 29 | Provide education geared to the population | 4,67 | 0,09 | |
| 39 | Ensure workers have sufficient means | 4,17 | 0,00 | |
| 59 | Directors must help the population without discrimination | 4,00 | 0,15 | |
| 35 | Activities must be managed transparently | 3,67 | 0,13 | |
| **CLUSTER 3** | **Mean of cluster** | **3,79** | **0,13** | |
| 2 | Do not cheat on each other | 4,00 | 0,12 | |
| 9 | Avoid social discrimination | 4,33 | 0,09 | |
| 53 | Decisions should be taken collegially | 3,67 | 0,08 | |
| 6 | Say what is fair and what is not | 4,17 | 0,11 | To have integrity (burkindlim tallgo) |
| 54 | Do not count only on aid | 3,83 | 0,23 | |
| 7 | Increase integrity | 3,67 | 0,10 | |
| 20 | Aid must not be diverted | 2,83 | 0,19 | |
| **CLUSTER 4** | **Mean of cluster** | **3,60** | **0,31** | |
| 1 | There must be honesty | 5,00 | 0,57 | |
| 25 | Ensure everyone knows we are all on the same footing in terms of equality | 3,50 | 0,66 | |
| 26 | Bring literacy to the whole population | 4,50 | 0,23 | |
| 52 | The country's resources are distributed inequitably | 3,17 | 0,33 | Trust each other (D ko sid ne taaba) |
| 8 | Guide each other | 1,67 | 0,38 | |
| 56 | Put the right man in the right place | 4,17 | 0,17 | |
| 13 | Do not divert aid from its intended objective | 2,83 | 0,18 | |
| 41 | Do not profit from politics to bully others | 3,67 | 0,18 | |
| 46 | Politics must not divide the population | 4,50 | 0,18 | |
| 50 | Politicians should avoid electioneering | 3,00 | 0,18 | |
| **CLUSTER 5** | **Mean of cluster** | **3,56** | **0,10** | |
| 17 | Leaders exploit the illiterate | 3,00 | 0,01 | |
| 57 | Work together toward the country's development | 4,17 | 0,11 | Transparency is good (veenem yaa soma) |
| 48 | Local officials must transmit faithfully the concerns of their populations | 3,50 | 0,18 | |
| **CLUSTER 6** | **Mean of cluster** | **3,56** | **0,26** | |
| 11 | We must support each other in life | 3,50 | 0,35 | |
| 31 | Help the children of peasants pursue their education | 4,50 | 0,16 | |
| 43 | Responsibilities should be alternated | 2,00 | 0,42 | Mutual support (d teel taaba) |
| 21 | Aid must be used in accordance with its intended objectives | 3,50 | 0,16 | |
| 37 | Ensure beneficiaries receive the tools they need for their work | 4,17 | 0,17 | |
| 36 | Ensure projects are monitored until they are done | 3,67 | 0,27 | |
| **CLUSTER 7** | **Mean of cluster** | **3,53** | **0,23** | |
| 10 | Work together in honesty | 3,50 | 0,24 | |
| 44 | Jobs and aid should be granted without regard to political affiliations | 3,00 | 0,04 | |
| 16 | Directors must be frank with those they manage | 3,83 | 0,33 | Mutual help (d za taaba) |
| 28 | Directors must look after the concerns of the rural populations | 3,67 | 0,35 | |
| 45 | Funds received for electoral campaigns should be used as intended | 3,67 | 0,19 | |
| **CLUSTER 8** | **Mean of cluster** | **3,20** | **0,20** | |
| 15 | Help the directors succeed in the tasks they assume | 2,17 | 0,14 | |
| 51 | Directors need to know that their strength comes from the population | 4,50 | 0,23 | |
| 27 | Work with everyone, without discrimination | 2,50 | 0,21 | Need other (ned la a to tiim) |
| 30 | Seek employment for everyone | 2,83 | 0,24 | |
| 33 | Hand out aid in front of several witnesses | 4,00 | 0,20 | |
| **CLUSTER 9** | **Mean of cluster** | **2,71** | **0,71** | |
| 3 | Tell them honestly what is being said | 3,00 | 0,64 | |
| 23 | Help those in the greatest need | 1,83 | 1,00 | |
| 24 | Help each other to avoid personal frustrations | 1,83 | 0,82 | Come Spontaneously To Carry Help (D zoe taab kuunga) |
| 12 | Each person is his neighbour's remedy | 2,33 | 0,64 | |
| 55 | Plan for a good future for our children | 3,83 | 0,80 | |
| 19 | Raise the awareness of the general population | 2,33 | 0,51 | |
| 14 | Directors must not divert aid to themselves, to the detriment of their subjects | 3,33 | 0,83 | |
| 58 | Each member of every organization must carry out his mission properly | 3,17 | 0,47 | |
