# Supplementary figures and images for: Equity and health policy in Africa: Using concept mapping in Moore (Burkina Faso)
Source: BMC Health Serv Res. 2008 Apr 22;8:90. doi: 10.1186/1472-6963-8-90 (PMC2386119; doi:10.1186/1472-6963-8-90)

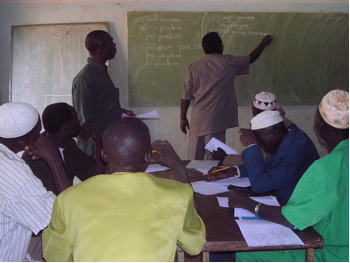

Supplement: Additional file 3 — Validation and labelling of the clusters in Moore. Picture of participants and moderators validating and labelling the clusters in Moore. [file 1472-6963-8-90-S3.png]

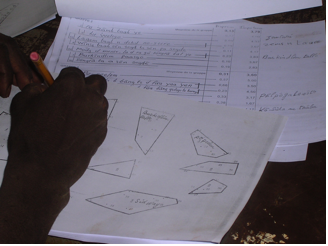

Supplement: Additional file 4 — Participant cluster labelling in Moore. Picture of one participant labelling clusters in Moore. [file 1472-6963-8-90-S4.png]
